# Novel molecular targets and natural drug candidates for the antifungal therapy of seborrheic dermatitis

**DOI:** 10.3389/fcimb.2026.1813026

**Published:** 2026-10-01

**Authors:** Viktor Filatov, Varvara Sekerina, Elena Kalenikova

**Affiliations:** Department of Pharmaceutical Chemistry and Organization of Pharmaceutical Business, Faculty of Medicine, Lomonosov Moscow State University, Moscow, Russia

**Keywords:** antifungal drugs, antifungal resistance, *Malassezia*, molecular target, natural products, seborrheic dermatitis

## Abstract

**Background:** The prevalence of seborrheic dermatitis (SD) involving scalp and non-scalp areas has spread worldwide because of the decreasing susceptibility to azole antifungal drugs and increasing diffusion of the multidrug-resistant fungi of *Malassezia* species. The antifungal resistance results from structural changes of well-known and new molecular targets and biochemical adaptations of fungi to the standard synthetical treatment. Meanwhile, the research and use of traditional plant products as an alternative therapy strategy for dermatological diseases has increased tremendously over the past last decade. This review is the first to systematically summarize the latest *in silico* and *in vitro* data of *Malassezia* molecular targets, natural products and the innovative insights for the development of new drugs against SD. After a meticulous screening of 6255 scientific literature records and 104 relevant studies on the selected topic, the sixth novel molecular targets have been found: carbonic anhydrase, lipases, sphingomyelinases, phospholipases and ABC-transporters in *Malassezia* strains and aryl hydrocarbon receptors (AhR) in host skin cells mediated various biological responses to *Malassezia* indole ligands. The eighteen plant extracts, essential oils and their constituents have been identified as potential candidates affecting these targets. Despite the promising potential of the described molecular targets and the natural drug candidates, there is a lack of clinical effectiveness, safety and pharmacokinetic data at that moment. Further longitudinal studies are necessary to elucidate the mechanisms involved in *Malassezia* resistance and determine whether natural products alone and in combination with clinically recommended drugs could contribute to SD prophylaxis and effective treatment.

## 1 Introduction

Abnormal peeling, itching, burning of the scalp, hair loss and excessive sebum production are all clinical symptoms of one of the most common dermatological diseases — seborrheic dermatitis (SD). Usually, the milder form of this disease is called dandruff involving non-scalp areas, such as the face, ears, upper chest, axillae, and inguinal folds. From 121 relevant articles, the global prevalence of diagnosed SD except dandruff is 4.38%, with significant heterogeneity across different ethnicities (Desai et al., 2022; Gupta et al., 2025). Meanwhile, the prevalence of dandruff caused by microbiota disbalance has been documented to range from 81% to 95% in African Americans, 66% to 82% in Caucasians, and 30% to 42% in Asian individuals (Gupta et al., 2025). Although the exact cause of SD has not definitely been established, it is believed that various factors can influence the manifestation of the disease. Internal factors include reduced overall biodiversity of the scalp microbiota and disrupted network connectivity between microbial strains, metabolic and hormonal disorders, skin barrier deterioration, immunity disorders, and changes in sebaceous gland secretion (Borda and Wikramanayake, 2015; Wasim et al., 2026; Yoon et al., 2026). External factors, such as acute and long-term stress, improper use of antimicrobial drugs and cosmeceuticals, poor hygiene of the scalp can exacerbate the scalp hygiene and provoke SD incidence (Borda and Wikramanayake, 2015; Boxberger et al., 2022; Sowmya et al., 2023). Undoubtedly, in addition to pathological manifestations, SD also has a significant psychological impact on the well-being of patients (Chen et al., 2025).

Several actual research results confirm that the severity and severe clinical manifestations of SD are associated with significantly lower fungal and bacterial diversity on human scalp (Carmona-Cruz et al., 2022; Gupta et al., 2026; Liang et al., 2025). The presence of microbial community consisting of *Staphylococcus epidermidis* (*S. epidermidis*), *Staphylococcus aureus* (*S. aureus*), and *Cutibacterium acnes* (*C. acnes*) strains is normally crucial for healthy scalp (Grimshaw et al., 2019). The commensal *C. acnes* is involved in reducing the loss of transepidermal water (TEWL), as well as maintaining an acidic pH on the surface of the scalp (Grimshaw et al., 2019). Meanwhile, fungal community based on *Malassezia* lipophilic yeasts is found on the skin of 75% to 98% of healthy adults (Piacentini et al., 2025; Kamamoto et al., 2017). Although the genus *Malassezia* comprises 18 recognized species, with at least 10 isolated from human skin, the species most consistently and frequently associated with SD are *M. restricta*, *M. globosa*, and *M. furfur* (Honnavar et al., 2024). *Malassezia* affects lipid-rich areas, using sebum components for metabolism, uses saturated fatty acids and ignores unsaturated fatty acids (as oleic acid), which leads to the development of inflammation (Carmona-Cruz et al., 2022; Piacentini et al., 2025). In case of diagnosed SD, an increase in the ratio of *Malassezia restricta/Malassezia globosa* (*M. restricta/M. globosa*) and a decrease in the ratio of *Cutibaterium/Staphylococcus* have been observed in clinical studies (Xu et al., 2016; Tao et al., 2021).

Antimicrobial resistance among *Malassezia* spp. is an emerging clinical concern that may significantly compromise the efficacy of standard antifungal therapies for SD (Galvis-Marín et al., 2025; Leong et al., 2025; de Pádua Oliveira et al., 2026). *Malassezia* fungi have adapted to some of the common antifungal agents and developed novel resistance mechanisms (**Figure 1**) to the most widely used class of antifungal agents is azoles, especially triazoles (Zheng et al., 2024). They are used as first-line drugs because of a wide spectrum of action, high efficacy, and a good safety profile (Zheng et al., 2024). Remarkably, 64.1% of isolated from patients with SD and identified *Malassezia* strains have been shown to be resistant to fluconazole *in vitro* (Wang et al., 2020). The research of 26 Colombian isolates of *Malassezia* spp. from patients with SD showed that the percentages of resistant fungal strains in the planktonic form were 42% for voriconazole, 31% for fluconazole, 23% for itraconazole, and 15% for ketoconazole (Galvis-Marín et al., 2025). Similarly, a decrease in sensitivity to azoles has been observed in 5 of the 26 isolates of *M. furfur* (Leong et al., 2021). To be adapted to ketoconazole and other azoles, resistant *Malassezia* strains increase the expression of efflux pumps such as ABC-transporters and pleiotropic drug transporters as well as other fungal targets like CYP51 (Park et al., 2020; Monk et al., 2020; Harris et al., 2021). The number of genes involved in ergosterol synthesis and mitochondrial iron metabolism also increases (Park et al., 2020). The overexpression of pleiotropic drug transporters genes as PDR10 gene is responsible for increased azole drugs efflux, like clotrimazole, miconazole and especially fluconazole (Leong et al., 2021). Despite genetic alterations, biofilm formation by *Malassezia* species also reduces their susceptibility to azole antifungals, necessitating substantially higher drug concentrations to achieve control of the SD, and contributes to the presence of persister cells, exhibiting resistance to some azoles in their mature state (Galvis-Marín et al., 2025; Čonková et al., 2022). The endpoint clinical efficacy of azole-based therapies depends on sum of approaches targeted on molecular pathways and antibiofilm activity of new drug candidates.

**Figure 1:**
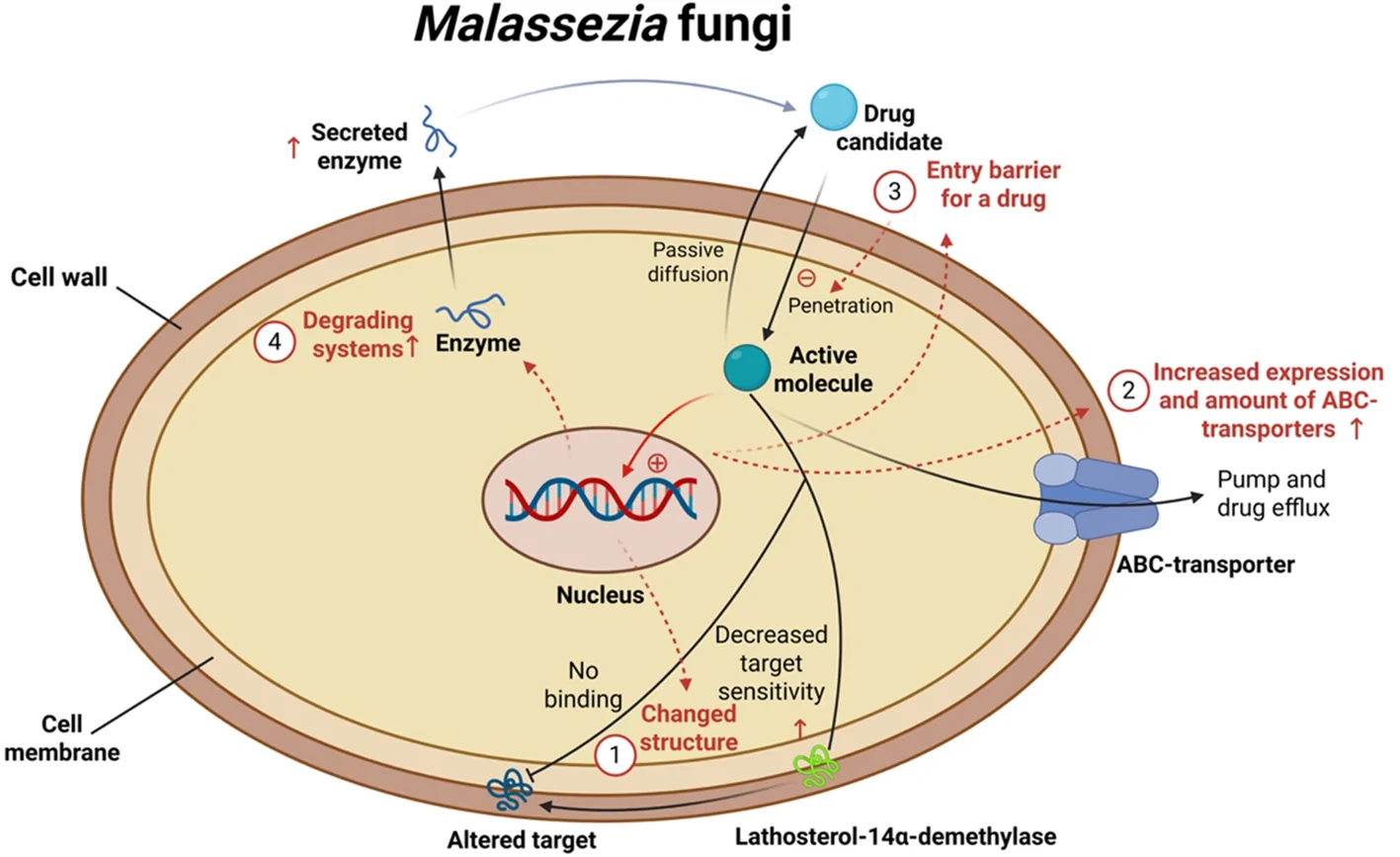
Molecular resistance mechanisms of *Malassezia* fungi cells to classical synthetic drugs.

Other non-azole substances such as piroctone olamine (Piquero-Casals et al., 2019), selenium disulfide (Turchin et al., 2025) and zinc pyrithione (Mangion et al., 2023) used in topical pharmaceutics and cosmetic shampoos exhibit antibacterial, antifungal, anti-inflammatory and keratostatic activity with accumulation of positive effects on scalp. However, the beneficial effects require improved transepidermal delivery because of low penetration of hydrophilic substances and skin exfoliation during washing process. Toxicological evaluation of zinc pyrithione reveals concentration-dependent adverse effects in human lymphocytes with induced oxidative stress, reduce cell viability, and cytostatic effects (Güner and Süleyman, 2020). Meanwhile in human keratinocytes, it triggers heat shock protein upregulation and DNA damage (Lamore and Wondrak, 2011). Long-term use of non-azole drugs leads to skin side effects including skin atrophy and discoloration, telangiectasia, hypopigmentation, skin infections, itching, skin dryness, and contact dermatitis (Del Rosso, 2011; Naldi and Rebora, 2009; Okokon et al., 2015).

The progressive emergence of antifungal resistance in *Malassezia* species, driven by genetic mutations (e.g., ERG11), gene multiplication, and efflux pump overexpression, is increasingly recognized as a significant factor compromising the efficacy of standard azole-based therapies and determining the likelihood of successful treatment outcomes in SD. Meanwhile, the growing interest in natural medicines could open new approaches for successful SD treatment worldwide (Javier Rodríguez Villanueva et al., 2018). Plants, namely plant extracts and essential oils, contain natural antifungal compounds and possess a number of biologically active substances that can serve as more effective and safer substances for the treatment of SD with possible lower risk of antimicrobial resistance of *Malassezia* species (Filatov et al., 2023). Currently, the investigation of novel plant-derived substances is conducted only *in silico* and *in vitro* without extended clinical trials. Therefore, this review first discusses novel molecular targets of *Malassezia* species as well as well-known targets, followed by an evaluation of natural product candidates based on *in silico* and *in vitro* data, and conclude with use limitations and future perspectives for the clinical evaluation of these drug candidates for SD treatment.

## 2 Molecular targets in *Malassezia* and their natural drug candidates

The phytochemicals from traditional remedies and medical plants are investigated for antibacterial and antifungal substances for prophylaxis and treatment of dermatological diseases. These molecules and complex substances have rich chemical composition, various pharmacological activities helpful for SD alleviation, good dermatological tolerance and higher safety on skin cells. One of the most advanced approaches is application of essential oils enriched by monoterpenes, sesquiterpenes, aromatic and aliphatic compounds. These active phytochemicals provide antibacterial (Jain et al., 2022), antifungal (Ayatollahi et al., 2021), anti-inflammatory (Caldefie-Chézet et al., 2004), antioxidant (Miguel, 2010) and regenerative (Sharifi-Rad et al., 2017) effects. Moreover, terpenes from essential oils can inhibit the essential enzymes of fungi and bacteria metabolism (Jalendiran et al., 2026) and increase membrane permeability for antifungal drugs (Blanc et al., 2023; Kasputė et al., 2025) with fungi lysis. It has been clinically confirmed that medical shampoos with 5% of *Melaleuca alternifolia* essential oil improved the scalp health only among 41% of patients with mild and moderate dandruff in comparison with control group (Satchell et al., 2002), that is not enough for effective therapy without use of synthetic drugs.

To provide effective therapy with lower resistance development, deep research of novel molecular targets in *Malassezia* cells is being conducted for further high throughput screening of candidates. *Malassezia* species, being involved in the development of SD, have specific molecular pathways and biochemical metabolism because of scalp environment, interaction with scalp bacteria and immune cells. The treatment of *Malassezia*-associated diseases is complicated by biofilm formation and resistance to synthetic drug due to increased expression of ABC-transporters, and multiple mutations in lanosterol-14α-demethylase (ERG11/CYP51) gene (Leong et al., 2021; Muslimin et al., 2019). Among the most promising approaches for effective antifungal therapy are molecular targets related to cell wall integrity, essential enzymes involved in pH regulation and cellular metabolism, receptors affecting downstream pathways in skin cells and efflux transporters providing the antifungal resistance to azoles: lanosterol-14α-demethylase (CYP51), carbonic anhydrases, ABC-transporters, lipases, sphingomyelinases, phospholipases and AHR ligands (**Figure 2**).

**Figure 2:**
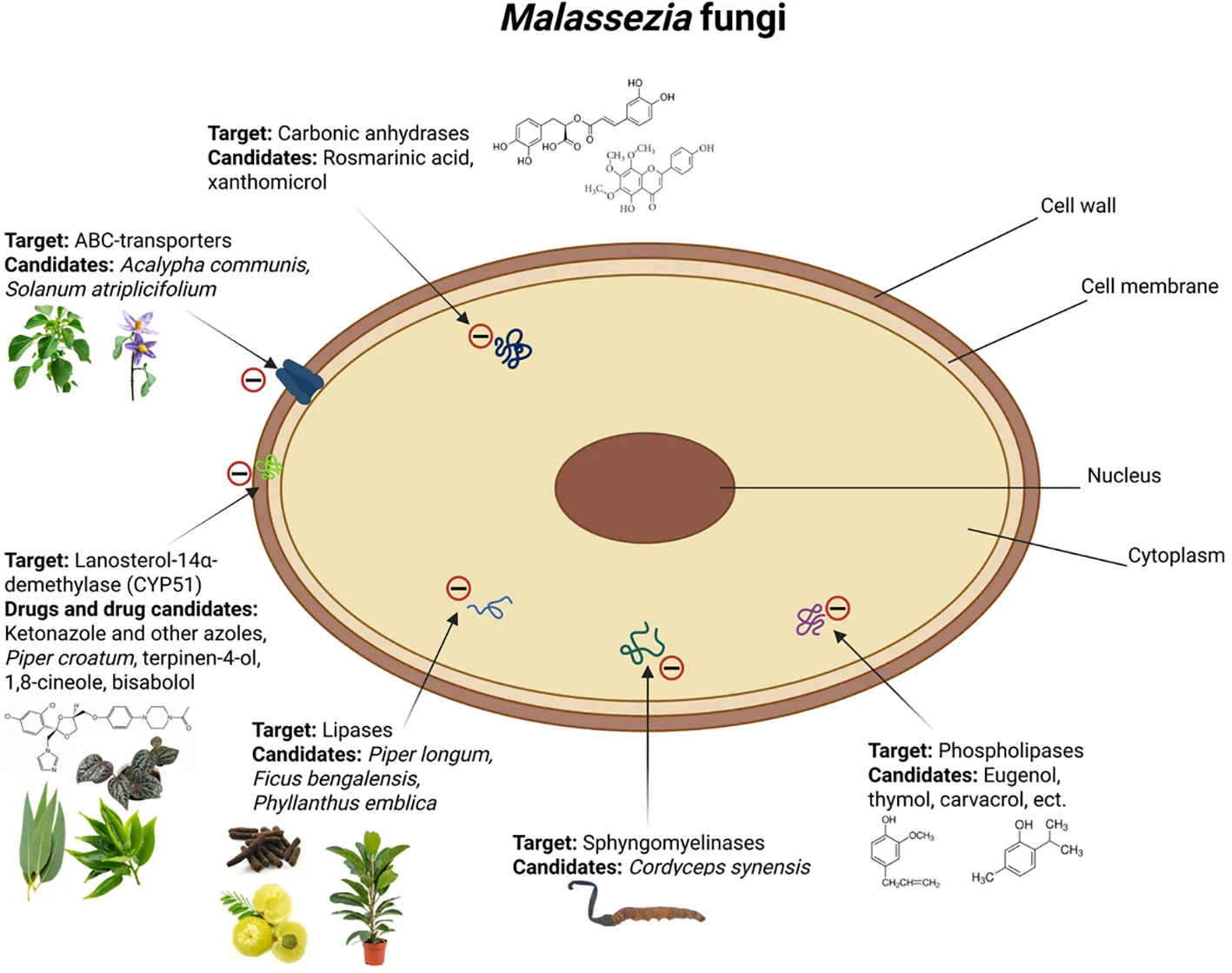
Molecular targets in *Malassezia* fungi and novel natural drug candidates: overview.

### 2.1 Lanosterol-14α-demethylase

Lanosterol-14α-demethylase (CYP51, Erg11, LDM) is one of the well-known identified targets of synthetic azole antifungal drugs (Hasanuddin et al., 2022; Monk et al., 2020). It is a cytochrome P450 enzyme that has activity in the growth process of fungal cell membranes. It regulates ergosterol synthesis by acting on one of the stages of its synthesis, the conversion of 4, 14-dimethylergostatrienol. Inhibitors of CYP51 disrupt its synthesis, thereby altering the structure of the cell membrane, leading to disruption of its integrity and permeability, and then to impaired growth and reproduction of fungal cells (Hasanuddin et al., 2022). The enzyme consists of several parts: catalytic C-terminal cytochrome P450 (CYP450) linked through an intermediate transmembrane helix (TMH1) to the N-terminal helix (MH1) which is located on the inner side of the endoplasmic reticulum (EPR) membrane, catalytic domain made of α-helices and β-layers, and also a heme residue. All these parts form the active center of the enzyme (Pochapsky, 2014; Sagatova et al., 2015). Azole drugs used in therapy contain an obligatory triazole or imidazole part (Zarei et al., 2025). The action of such azole drugs is based on coordination of one of the nitrogen in the azole ring as a sixth axial ligand with heme iron LDM, blocking activation by oxygen. The interaction with heme is a major determinant of azole drug binding to LDM, but it can also cause side effects by binding to host LDM or non-target cytochrome P450 enzymes required for drug elimination. However, azoles provide rather fungistatic than fungicidal therapy for common human fungal infections, and their efficacy in treating many of them is limited over time.

There are currently five types of therapeutic agents used worldwide to treat fungal infections. These include acrylamines, polyenes, echinocandins, azoles and antimetabolites. In addition to toxicity issues and drug interactions, the clinical efficacy of existing antifungals is limited by the growing problem of azole resistance, which stimulates the search for novel compounds targeting CYP51 (Zarei et al., 2025). It is noted that prolonged or excessive use of azoles leads to the development of fungal multi-resistance due to overexpression of CYP51s as well as the emergence of multiple analogs of CYP51 (Monk et al., 2020). The decrease in sensitivity of yeast-like fungi of the genus *Malassezia* to ketoconazole, miconazole, and clotrimazole is observed among patients with SD (Leong et al., 2021).

It is currently known that some biologically active natural products can be promising therapy of SD. The recent study based on *in silico* and *in vitro* has shown that combination of *Melaleuca alternifolia (M. alternifolia)* leaf oil, 1, 8-cineole and (-)-α-bisabolol has synergetic antifungal effect due to penetration enhancing and action on lanosterol-14α-demethylase (Filatov et al., 2023). The natural substance reduces the fungal growth of *M. furfur* with an average efficiency up to 99.85% *in vitro* (Filatov et al., 2024). Revealed efficiency exceeds the climbazole-mediated reduction and is comparable with ketoconazole efficiency *in vitro*, but further clinical evaluation on patients with SD is crucial in proof-of-concept of the hypothesis.

Another traditional natural product named *Piper crocatum* reduces reliance on synthetic antifungals and combats multi-resistant fungal strains. It is a plant from *Piperaceae* family, which has long been used in traditional medicine in India, China, and Korea to treat various human ailments (Siswina et al., 2022). *P. crocatum* extracts have been tested against *C. albicans*, showing MIC values in the range of 1.25–2.5% w/v (Siswina et al., 2022). This action is due to inhibition of the biosynthesis of ergosterol, specifically lanosterol-14α-demethylase. Among ten compounds isolated from the plant, a sesquiterpene glycoside named erigeside II (dictamnoside A) exhibits the strongest antifungal activity (Siswina et al., 2022) because of low molecular weight, sufficient topological polar surface area and eight hydrogen bond acceptor for binding in active site of lanosterol-14α-demethylase, still inferior to synthetic antifungal agents (Trösken et al., 2006). Considering the safety issues, *Piper crocatum* leaf extract enriched by phenolic acids and flavonoids has sufficient antioxidant activity using the 2, 2-azinobis(3-ethyl-benzothiazoline-6 sulfonic acid (ABTS) method (Pratami et al., 2025) and wound-healing effect, supporting the dermatological benefits on scalp and skin.

### 2.2 ABC transporters

ABC transporters or ATP-binding cassette transporters are a family of membrane transport proteins, efflux pumps, that bind to a wide range of substrates (Harris et al., 2021; Park et al., 2020). ABC transporters consist of two pairs of domains: a transmembrane domain (TMD) and a nucleotide-binding domain (NBD). Substrate passage through the lipid bilayer occurs by binding to ATP and its hydrolysis by the NBD, causing conformational changes in the TMD that allow transport of H^+^ ions, providing an electrochemical gradient to activate the transport cycle (Harris et al., 2021; Leong et al., 2021). These transporters remove toxic compounds from the cells, thus conditioning the development of multidrug fungal resistance. The yeast ABC transporters are subdivided into six subfamilies including up to 30 proteins. One of these subfamilies can be called the subfamily of pleiotropic drug resistance (PDR). They are characterized by the inclusion of a structural repeat that results in a reverse transmembrane topology compared to other ABC transporters. Pathologically relevant transporters of this subfamily are PDR5 and PDR10. PDR5 transports wide range of chemicals including azoles, ionophores, antibiotics and other xenobiotics (Harris et al., 2021). Moreover PDR10 is involved in fluconazole resistance in *M. furfur* (Leong et al., 2021).When azoles are investigated *in vitro*, PDR10 expression increases during 4 weeks of drug application and decreases after drug withdrawal. Other yeast ABC transporters, cerebellar degeneration related 1 (CDR1) and multidrug resistance (MDR) confer drug resistance to *C. albicans*. Thus, enhanced expression of ABC-transporters by pathogenic fungi is one of the factors of their decreased sensitivity to synthetical fungicidal drugs (Kano et al., 2019).

Available data *in vitro* indicates that non-toxic and highly active extracts from *Acalypha communis* and *Solanum atriplicifolium* offer a promising source of natural compounds for potentiating the antifungal effect of fluconazole by blocking MDR1 and CDR1 efflux transporters and decreasing impact of one of the antimicrobial resistance mechanisms (Gil et al., 2022). These extracts substantially reversed fluconazole resistance in *Candida* species. Among all phytochemicals isolated from the aerial parts of *Acalypha communis*, terpenoids, phenols and cycloartane-type triterpenes show activity against resistant gram-positive bacteria and dermatophyte strains (*Trichophyton rubrum*, *Trichophyton mentagrophytes*) as natural alternatives for treating superficial skin infections and candidiasis (Cappiello et al., 2020; Villaescusa-González et al., 2026). The *Acalypha communis* extract has high anthocyanin and antioxidant activity, higher than that of ascorbic acid by 3.5-fold (Abdelbaky et al., 2025). *Acalypha* species extracts generally demonstrate low cytotoxicity on HaCaT keratinocytes at lower concentrations while potentially protecting against oxidative stress, but additional research is needed to confirm safety for skin applications at appropriate concentrations (Seebaluck-Sandoram et al., 2018; Villaescusa-González et al., 2026). Additionally, the *Solanum* species natural products show the highest anti-inflammatory activity and cytotoxic potential on various cancer cell lines (Alsherbiny et al., 2015), but there is a lack of data of safety and cytotoxicity on skin cells. These plant extracts should be standardized to a single active compound to guarantee reproducible effects in preclinical and subsequent clinical studies.

### 2.3 Lipase

In patients with SD sebaceous glands are highly active, but the lipid film on the surface of the scalp is thickened. The lipid film consists of both lipids formed by sebocytes and secreted to the surface and lipids derived from keratinocytes that are incorporated into the layers of the stratum corneum of the epithelium (Adalsteinsson et al., 2020). *Malassezia* species yeasts parasitizing in SD lack genes encoding the fatty acid synthase, so they cannot independently synthesize fatty acids needed for growth (Čonková et al., 2024). Therefore, these fungi secrete hydrolases such as lipases, phospholipases, and acidic sphingomyelinases, that facilitate the acquisition of fatty acids from outside. Lipases are considered to be the main factor in the pathogenicity of *Malassezia* strains on scalp. They catalyze the hydrolysis of triacylglycerols to form free fatty acids, diacylglycerol, monoacylglycerol and glycerol. A prime example of a lipase is *M. globosa* LIP1 (SMG1), which is a monoacylglycerol (MAG) and diacylglycerol (DAG) lipase (Mishra et al., 2021). *Malassezia* utilizes saturated fatty acids and secrets irritating unsaturated fatty acids, such as oleic acid, which is a major trigger of inflammation in SD (Adalsteinsson et al., 2020). These newly formed fatty acids not only support yeast growth but also cause skin inflammation, flaking and itching (Billamboz and Jawhara, 2023; Mishra et al., 2021). Thus, SMG1 may be a promising target to inhibit fungal growth and control SD (Mishra et al., 2021; Guo et al., 2015).

As an example of targeted drug, propanediol caprylate (propylene glycol ester of caprylic acid) is an innovative antifungal substance whose mechanism of action is based on specific cleavage by *Malassezia* lipases. This ester, structurally similar to sebum triglycerides, is rapidly hydrolyzed by extracellular fungal lipases and hydrolases to release free caprylic acid with strong antifungal effect (Mayser et al., 2024). The key feature of this mechanism is that the released caprylic acid exerts a self-regulating (“auto-kill”) effect on the *Malassezia* population: the higher the density of fungal cells, the more ester is hydrolyzed and the stronger the antifungal effect. Importantly, this mechanism remains effective even against azole-resistant strains, as demonstrated in studies on *M. pachydermatis* (Koch et al., 2022).

Meanwhile, there are some natural phytochemicals that can be used as *Malassezia* lipase inhibitors. Namely, for the extract of *Piper longum (Linn.)* the percentage of lipase inhibition is up to 35.32% in a dose-response relationship according to *in vitro* research results (Gomare et al., 2018), requiring the use of combined therapy with clinically approved drugs for SD. *Piper longum L.* is widely distributed in the Indian subcontinent, Sri Lanka, Middle Eastern countries and the USA. *P. longum L*. has been used in traditional remedies as well as in the Ayurvedic system of medicine against various disorders (Biswas et al., 2022, Biswas et al., 2024). Additional to antifungal activity, ethanolic *Piper longum* root, fruit, leaf extract enriched by alkamide compounds exhibits potent antibacterial activity against biofilm-forming multidrug-resistant *Staphylococcus aureus* and anti-inflammatory effect through inhibition of matrix metahoproteinases-8 and inducible nitric oxide synthase (iNOS) in skin cells (Gaur et al., 2025). The phytochemical complex of piperine, piperlongumine, sylvatin, sesamin, diaeudesmin, piperlonguminine, pipermonaline, and piperundecalidine in *Piper longum* extract involves the activation of cellular and humoral immune responses for the treatment of dermatological diseases with good safety profile (Osman gani et al., 2019).

Also, the inhibitory activity has been found in *Ficus benghalensis (Linn.)* aerial roots extract as 32.18%, and for *Phyllanthus emblica (Linn.)* as 26.62%. The lipase activity of *M. furfur* is dependent on the pH of the environment, cations present in the cell cytoplasm, sebum substrates as well as dosages of added drugs. These inhibitory effects of plant extracts open up the new possibility to control *Malassezia* species on human scalp through newer and safe herbal formulations over synthetic drugs (Gomare et al., 2018). To improve the skin condition during SD, *Ficus benghalensis (Linn.)* extract containing a wide range of phytoconstituents, including phenols, flavonoids, alkaloids, tannins, saponins, terpenoids, glycosides, and steroids displays a significant antioxidant capacity higher than ascorbic acid using DPPH and FRAP methods and accelerates wound healing through a decreased epithelization period (Murugesu et al., 2021). The *F. benghalensis* root extract significantly improves collagen synthesis in dermis that could be helpful for normalization of scalp microenvironment (Singh et al., 2023; Logesh et al., 2023). The *Phyllanthus emblica (Linn.)* fruit extract mitigates skin aging process in fibroblasts through a targeted influence on aquaporins 3 type for skin barrier restoration and sebum regulation helpful for SD and dandruff management (Meng et al., 2025; Timudom et al., 2020). These plant extracts should be standardized to a single active compound to guarantee reproducible effects in dose-dependent manner preclinical and subsequent clinical studies.

### 2.4 Phospholipase

*Malassezia* produces many extracellular phospholipases, which are considered pathogenic factors (Billamboz and Jawhara, 2023). Phospholipases (PLs) are a heterogeneous group of enzymes that catalyze the hydrolysis of glycerophospholipids by cleaving one or more glycerol-phospholipid ester bonds, resulting in the release of free fatty acids that cause inflammation in the host body, such as oleic acid and arachidonic acid. Oleic acid damages keratinocytes provoking skin flaking. The breakdown products of arachidonic acid can damage the outer layer of the skin, disrupt the skin barrier and lead to abnormal keratinization of the skin (Galizia et al., 2024). To date, there is no information on the effect of widely used antifungal drugs on the production of extracellular phospholipase, which is also a promising target for SD therapy. For example, components of some plant essential oils not only have antifungal properties but also affect the production of extracellular phospholipase by *Malassezia* strains (Billamboz and Jawhara, 2023; Čonková et al., 2024; Park et al., 2020).

There is an *in vitro* study aimed at investigating the inhibitory effect of selected azole antifungals (itraconazole, posaconazole and voriconazole) and plant essential oil components (terpinen-4-ol, thymol, carvacrol, eugenol and geraniol) on fungal phospholipase, evaluated by Egg Yolk Agar plate method (Čonková et al., 2024). After one-hour exposure to plant essential oil components, the best inhibitory effect was recorded for eugenol (62.3%), followed by terpinen-4-ol and thymol (56.6%), geraniol (41.5%) and carvacrol (26.4%). A three-hour exposure revealed that thymol retained the best inhibitory effect (88.7%) on phospholipase production, followed by carvacrol (73.6%), eugenol (56.6%), terpinen-4-ol (52.8%) and geraniol (49.1%). After six-hour exposure, no growth of *M. pachydermatis* strains exposed to carvacrol was observed, and the inhibitory efficiency for the other tested essential oil components achieved 88.7%. The results were compared with percentages of inhibitory efficacy on extracellular phospholipase with known antifungal drugs such as itraconazole with 26.4% (one-hour exposure), 47.2% (three-hour exposure) and 50.9% (six-hour exposure), posaconazole (26.4% for one-hour, 28.3% three-hour and 28.3% six-hour exposure) and voriconazole (18.8% one-hour, 20.8% three-hour and 35.8% six-hour exposure) efficacy. This investigation showed the prospect of several natural essential oils standardized for eugenol, terpinene-4-ol, thymol, geraniol and carvacrol in the treatment of SD. Despite these encouraging *in vitro* findings, the ultimate clinical efficacy of the essential oil components may be substantially compromised by the complex scalp microenvironment, which is characterized by sebum overproduction, pH fluctuations, biofilm formation, and interactions with commensal microorganisms. In addition, translational hurdles such as limited skin bioavailability, poor formulation stability, and a lack of standardized clinical trials further pose additional challenges that must be overcome before these natural compounds can be reliably translated into clinical practice.

According to recent data, eugenol, as a component of essential oils, exhibits anti-inflammatory, analgesic, antimicrobial and antioxidant properties, as well as enhances skin penetration of various drugs (Nejad et al., 2017; Tariq et al., 2025; Qureshi et al., 2025; Damasceno et al., 2024; Tavvabi-Kashani et al., 2024; Schafer et al., 2023) because of activation of various biological pathways. Thymol, as the main active phytochemical responsible for the activity of thyme essential oil, has been shown to possess antibacterial effect against biofilms of *Staphylococcus aureus* (Kowalczyk et al., 2020). Carvacrol, either alone or in combination with other compounds, exhibits a strong antimicrobial effect on skin-related bacteria and anti-inflammatory modulation by inducing antioxidant protection systems and reducing the level of pro-inflammatory cytokines in skin cells (Mączka et al., 2023). Considering contact allergenic properties of essential oils and their molecules by cosmetic industry under the EU Cosmetics Directive, there is a recommendation to limit applied concentrations of eugenol, thymol and carvacrol alone or in the composition of essential oils in products that remain on the skin and scalp for long-term period.

### 2.5 Sphingomyelinase

Normally, a healthy microbiome produces metabolites necessary to maintain skin moisture, elasticity and firmness, including the synthesis of ceramides by sphingomyelinase. Thus *S. epidermidis* stimulates the production of ceramides in the skin by producing the enzyme sphingomyelinase, which hydrolyzes sphingomyelin present in skin cell membranes, consequently releasing ceramides, an essential component of the lipid matrix of the stratum corneum. Ceramides maintain the osmotic barrier of the skin, thereby improving hydration, preventing TEWL (Theodorou et al., 2024) and promoting keratinocyte differentiation (Klas et al., 2021). In SD, there is a dramatic change in the levels of bioactive sphingolipids, such as ceramides (Klas et al., 2021). The *Malassezia* yeasts produce acidic sphingomyelase, and it similarly hydrolyzes the membrane lipid sphingomyelin to ceramide and phosphorylcholine. This leads to excessive production of ceramides and promotes the growth of *Malassezia* (Billamboz and Jawhara, 2023).

Speaking about natural acidic sphingomyelase inhibitors, *Cordyceps sinensis* (CS) extract represents a promising natural medicine drug. A parasitic fungus known as *Cordyceps* has long been coveted as an exotic traditional Chinese medicine with reported pharmacological applications, including immune, kidney, respiratory, lung and cardiovascular diseases (Wu et al., 2022; Chen et al., 2013). The caterpillar fungus *Cordyceps sinensis (Berk) Sacc.* stands out as the most documented species of *Cordyceps* with reports documented in Tibetan medical texts since 15th century (Chen et al., 2013). Cordycepin, cordycepic acid, cordyglucan, cordymin, adenosine are potent bioactive metabolite of CS extract known for various pharmacological properties, such as anti-inflammatory, immunomodulatory, antioxidant and anti-allergic effects for skin and scalp health (Ashraf et al., 2020). *Cordyceps* mycelia extract (CME-1) is a promising source of therapeutic medicines because it can be mass-produced and it has a chemical composition similar to that of the fruiting body of CS (Yuan et al., 2022; Chen et al., 2024; Lee et al., 2020). CME-1 inhibits neutral and acid sphingomyelinases in a concentration-dependent manner, with a half-maximal inhibitory concentration (IC50) of approximately 50 μg/mL (Yuan et al., 2022; Chen et al., 2024; Lee et al., 2020). Despite the general recognition of the *Cordyceps sinensis* extract as safe for cosmetic use over the past three decades, the isolated bioactive components, cordycedipeptide A and cordyceamides A and B, have been shown to exhibit significant cytotoxic activity on mammalian cells (Liu et al., 2015). This finding underscores the critical necessity for rigorous extract standardization and thorough post-extraction purification protocols to ensure product safety and biocompatibility.

### 2.6 Carbonic anhydrase

During their life cycle fungi are exposed to various environmental factors, including changes in the concentration of carbon dioxide (CO_2_) (Nocentini et al., 2017). The concentration of CO_2_ present in mammalian tissues is about 5%, compared to 0.03% in atmospheric levels. This difference is captured by specific adaptation of fungi (CO_2_ sensing) (Supuran and Capasso, 2021), which ultimately stimulates colonization of the host’s organism by pathogens (De Luca et al., 2021). CO_2_ can potentially act as an activator of several signaling pathways in cells, such as affecting the triggering of cells differentiation and biosynthesis of proteins involved in the metabolism and pathogenicity of these microorganisms. The molecular mechanisms involved in sensing CO_2_ represent a promising target for the development of antifungal drugs (De Luca et al., 2021). Carbonic anhydrase (CA) is a metalloenzyme that catalyzes the reversible reaction of carbon dioxide hydration to bicarbonate and proton (CO_2_ + H_2_O⇋ HCO_3_^−^ + H^+^) (Nocentini et al., 2017). In this way, bicarbonate is produced and acts as a promoter of fungal meiosis and sporulation. Adenylate cyclase (AC) is directly activated by bicarbonate (Supuran and Capasso, 2021), then its activity as well as concentration of cyclic adenosine monophosphate (cAMP) is increased, leading to stimulation of the development of filamentous structures (hyphae) necessary for fungal virulence, adhesion and hydrolase production. These biochemical changes cause cell death in the colonized host. Eight families of carbonic anhydrases found in different organisms are: α-, β-, γ-, δ-, ζ-, η-, θ-, and ι -Cas (De Luca et al., 2021). So, in the fungus genus *Malassesia*, β-CA or MgCA (*M. globsa*) is represented, which occurs with at least one isoform. In contrast, α-CA is rarely found in fungi (Nocentini et al., 2017; De Luca et al., 2021). Synthetic inhibitors of this enzymes include inorganic anions, phenols, polyamines, dithiocarbamates, xanthates, monothiocarbamates, and sulfonamides (Nocentini et al., 2017; Supuran and Capasso, 2021).

Natural phenolic substances with inhibitory activity can be found in plants, such as xanthomichol and rosmarinic acid from *Origanum dictamnus L., Thymus vulgaris L. and Rosmarinus officinalis* (Paloukopoulou et al., 2022; Köksal and Şenol, 2025). The phytochemicals selected from these plants belong to famous flavonoids chemical family. Additionally, they include monoterpene dimers, such as the new natural product I4-II7-di-carvacrol, methoxylated flavones, one neolignane and rosmarinic acid (Paloukopoulou et al., 2022). The methoxylated flavone xanthomicrol (Ki = 0.6 μM) and rosmarinic acid (Ki = 2.2 μM) have been found to selectively inhibit *Malassezia* globosa carbonic anhydrase at submicromolar concentrations, demonstrating substantially higher potency than the standard inhibitor acetazolamide (Ki = 40.0 μM) (Paloukopoulou et al., 2022). These compounds are not cytotoxic, but show no activity against *M. furfur* strain (Paloukopoulou et al., 2022). It is likely that flavonoid natural products have strain specificity attributed to the relatively low sequence homology of carbonic anhydrase across different *Malassezia* species, as demonstrated for *M. globosa* (MgCA) and *M. restricta* (MreCA) (Del Prete et al., 2019; Del Prete et al., 2020).

Rosmarinic acid is an ester of caffeic acid and 3, 4-dihydroxyphenyllactic acid, and it can be found in herbs of different plant families, especially *Lamiaceae*. The presence of rosmarinic acid in medicinal plants has a number of interesting biological activities, such as antiviral, antibacterial, anti-inflammatory and antioxidant. In plants, rosmarinic acid is supposed to act as a preformed constitutively accumulated defence compound (Harindranath et al., 2025; Guan et al., 2022; Jakovljević et al., 2025). Moreover, this substance has demonstrates a favorable safety profile to human keratinocytes at concentrations up to 100 μg/mL (Koycheva et al., 2024). It shows promising potential for clinical use by improving skin barrier function and reducing inflammation in conditions such as SD, as evidenced by a randomized controlled trial where a topical rosemary extract lotion reduced symptoms comparable to 2% ketoconazole lotion (Sadati et al., 2024).

## 3 Aryl hydrocarbon receptor ligands from *Malassezia* fungi

The aryl hydrocarbon receptor (AHR), also known as the dioxin receptor, is localized in the cytoplasm of various cell types, including keratinocytes, sebocytes, fibroblasts, dendritic cells, and T-lymphocytes in the skin, where it acts as a sensor mediating the regulation of immune and inflammatory responses (Fernández-Gallego et al., 2021). Given the central role of immune cells and inflammation in the pathogenesis of SD, this receptor may be involved in the mechanisms underlying progression by modulating local immune responses and inflammatory processes in the skin.

The aryl hydrocarbon receptor is a ligand-dependent transcription factor. It can be activated by a variety of exogenous and endogenous environmental factors, including polycyclic aromatic hydrocarbons (PAHs), halogenated aromatic hydrocarbons (HAs or dioxins), and metabolites derived from catabolism of L-tryptophan (L-Trp). This suggests that AHR contains a relatively promiscuous ligand binding site. Ligand binding in the cytosol induces the synthesis of enzymes of the cytochrome P450 (CyPs) family, namely CYP1A, CYP1A2 and CYP1B1. CYP450 enzymes are involved in the metabolism of xenobiotic compounds, for example most of the environmental carcinogens acting on the skin require metabolic activation (Fernández-Gallego et al., 2021; Gaitanis et al., 2011).

In addition, AHR activation is an important regulator of skin barrier function, involved in detoxification and pigmentation, cellular homeostasis, control of immune-mediated skin responses and the production of skin barrier proteins *in vivo* and *in vitro* for epidermal terminal differentiation of keratinocytes. All this is due to the presence of endogenous, non-xenobiotic ligands (Fernández-Gallego et al., 2021). In studies conducted in mice with targeted ablation of *Arnt (AHR nuclear translocator)* in keratinocytes, the mice exhibited severe defects in exfoliation and epidermal barrier function. These data indicate that specific alterations of AHR function in keratinocytes may lead to enhanced barrier damage and facilitate the penetration of bacterial microbiota. On the other hand, unregulated AHR signaling in the skin can trigger allergic inflammation by inducing dysbiosis of the skin microbiota (Fernández-Gallego et al., 2021; Billamboz and Jawhara, 2023).

One type of ligand is the group of L-tryptophan (L-Trp) metabolites. They can be a product of the life activity of opportunistic skin bacteria or fungi. Thus, the cutaneous eukaryotic mycobiota is dominated by the family *Malasseziaceae. Malassezia* species produce some potent indole ligands: indolo[3, 2-b]carbazole, malassezin, indirubin, pityriacitrin, pityrazepine and 6-formylindolo[3, 2-b]carbazole (**Figure 3**). In addition to the effects described above, the binding of these ligands to the receptor also increases levels of Toll-like receptor 2 (TLR-2), IL-1β, IL-6, IL-8, IL-17, IL-19, IL-22, IL-23, and cyclooxygenase 2 (COX-2). Therefore, activation of AHR by malassezia-derived agonists may lead to the exacerbation of pathologic skin conditions (Fernández-Gallego et al., 2021; Billamboz and Jawhara, 2023).

**Figure 3:**
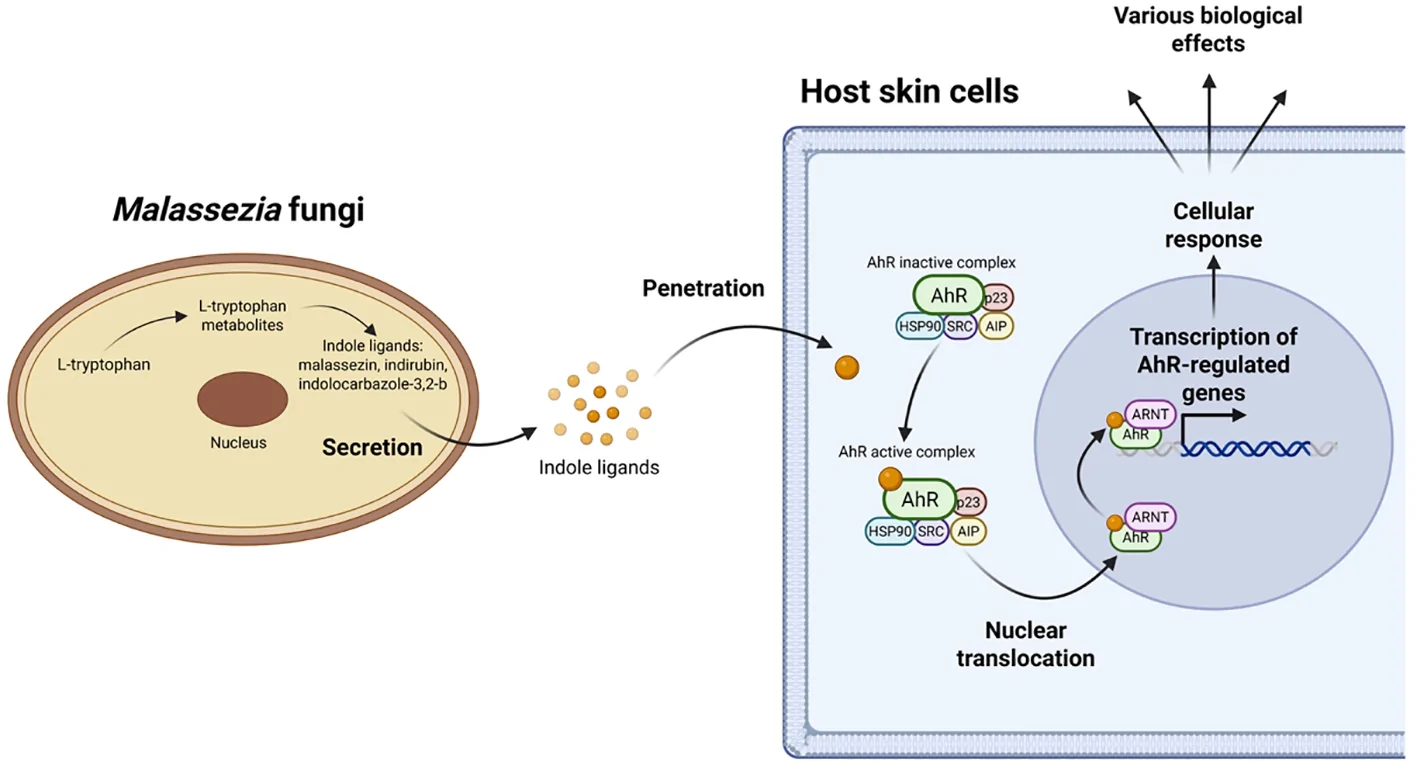
Activation of AHR. The cutaneous *Malassezia* yeast has the ability to synthesize ligands AHR [indirubin (ind), indolo[3, 2-b]carbazole (ICZ), malassezin] that can activate AHR and affect downstream pathways.

A great prospect in the treatment of SD is the use of *Rosemary* phytochemicals. It is a shrub of the *Lamiaceae* family, which has been widely used in the Mediterranean area since antiquity in folk medicine and as a culinary spice. Almost all over the world, *Rosemary* leaves are used as decoctions, infusions and tinctures to cure or prevent a wide range of health disorders (Ribeiro-Santos et al., 2015). The main isolated metabolites of this plant are 4, 7-*O*-dimethylapigenin, 7-*O*-methyl-epi-rosmanol, carnosol, carnosic acid and betulinic acid (Kallimanis et al., 2022). Five different dry *Rosmarinus officinalis* L. extracts (ROEs) show similar dose-dependent activities with almost complete inhibition of AHR activation by ligand of 2, 3, 7, 8-tetrachlorodibenzo-*p*-dioxin (TCDD) at 100 ppm. One of ROEs at 10 ppm shows 99%, 50%, 90%, and 85% inhibition against such ligands of microbial origin as 6-formylindolo[3, 2-b] carbazole (FICZ), indirubin (IND), pityriazepin (PZ) and 2, 3, 7, 8-tetrachlorodibenzo-*p*-dioxin (TCDD) in human keratinocytes model. Most assayed metabolites exhibit dose-dependent antagonist activity. ROEs inhibit AHR activation by TCDD and by the malassezia metabolites FICZ, PZ, and IND. Hence, ROEs could be useful for the prevention or treatment of skin diseases mediated by activation of AHR, especially in presence of SD (Kallimanis et al., 2022). Being helpful for SD management, *Rosmarinus officinalis L*. extracts have been used in topical pharmaceutics and cosmetic products for its antioxidant, anti-inflammatory, antibacterial, antinociceptive and wound healing properties attributed to the presence of phenolic acids and flavonoid secondary metabolites in normal and disease-induced models like atopic dermatitis (Macedo et al., 2020; Rahbardar et al., 2017). Moreover, rosemary extract shows significant hair growth after the 16th day of treatment compared with those in the control because of modulation of inflammatory cytokines, antioxidant protection of hair follicles and inhibition of 5α-reductase (Choi et al., 2024; Meziane et al., 2025; Allam et al., 2025).

## 4 Materials and methods

Relevant scientific original articles and reviews were searched in the electronic databases Google Scholar and PubMed, as well as on the publishing platforms of Frontiers, ScienceDirect, MDPI, and Wiley Online Library. The search covered the period from 2010 to 2025 (the last 15 years), with particular emphasis on works published within the last two years to incorporate the most recent data. The search included the following keywords and their combinations: “seborrheic dermatitis therapy”, “dandruff”, “aryl hydrocarbon receptor”, “seborrheic dermatitis Malassezia”, “antifungal targets”, “seborrheic dermatitis in adults”, “Malassezia globosa lipase”, “dandruff targets”, “Malassezia AHR”, “seborrheic”, “ICZ”, “Malassezia”, “sebocytes”, “AHR in keratinocytes”, “lanosterol-14α-demethylase”, “abc-transporters”, “phospholipids”, and “sphingolipids”. The literature selection process is presented as a PRISMA flowchart (**Figure 4**). Initially, 6255 records were identified: 6110 from Google Scholar and 145 from PubMed. After removing duplicates (n=138), 6117 records remained for screening by title and abstract. At the title/abstract screening stage, 5737 records were excluded as not relevant to the review topic. The remaining 380 publications were sought for full-text retrieval, of which 18 could not be obtained. Based on the full-text reading of 362 articles, 258 records were excluded for various methodological reasons described in **Figure 4**. Ultimately, 104 studies were included in the final analysis and reference list and were incorporated into the narrative review. All selected *in vitro* and *in silico* studies were thoroughly analyzed with a focus on well-known and novel molecular targets in *Malassezia* fungi, mechanisms of action of natural products, data on antimicrobial resistance, and dermatological safety. The main statements presented in the review, including numerical values and statistical criteria, are based on the results reported in these sources.

**Figure 4:**
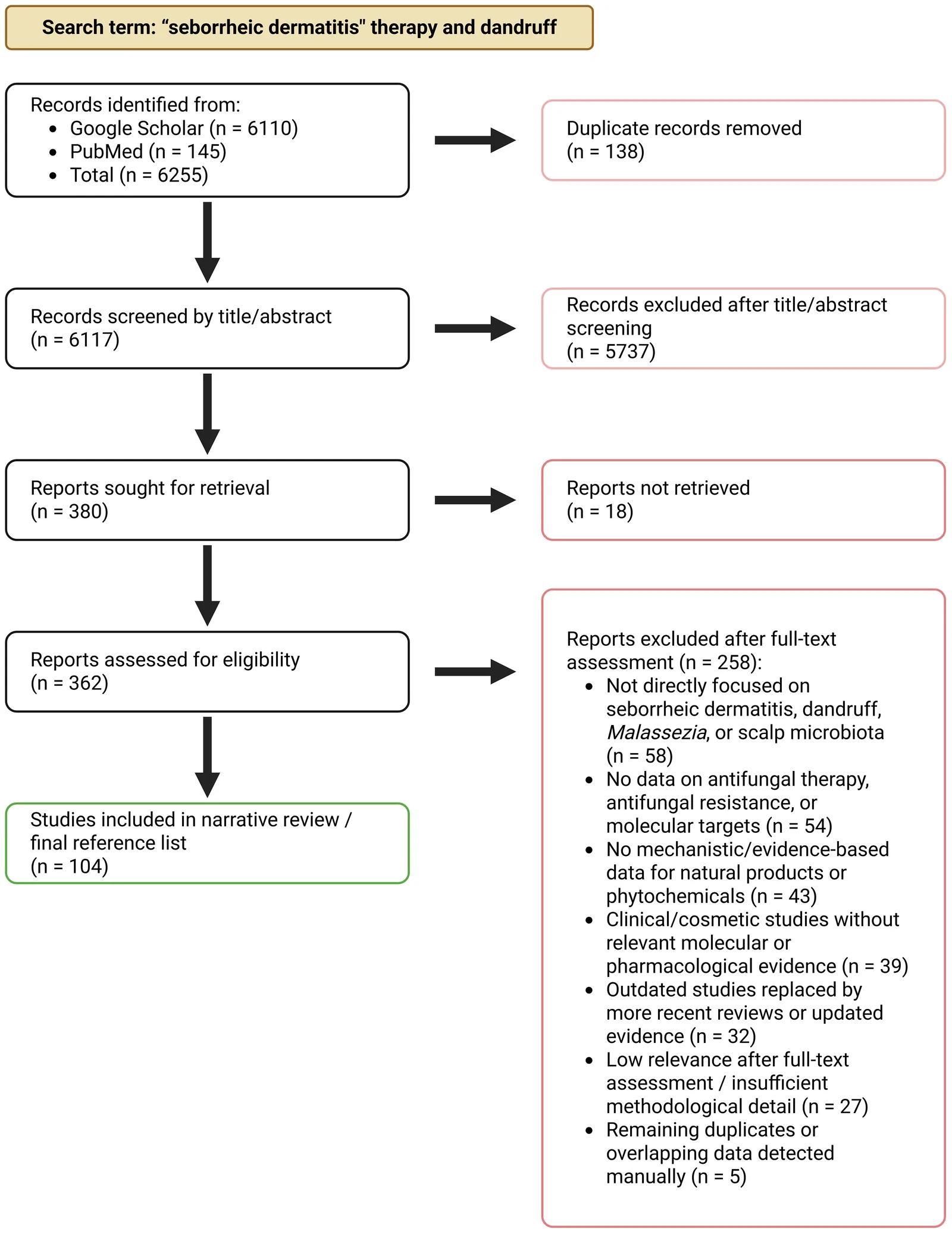
PRISMA flowchart of study selection.

## 5 Conclusion and recommendations

The global prevalence of SD because of multi-resistant *Malassezia* strains is currently a highly relevant problem in dermatology. Despite use of azole synthetic drugs as a gold standard therapy, there is a lack of new approved antifungal drugs for successful antifungal treatment of SD. Issues relating to adverse reactions to antifungal therapy in recent times are also becoming more vivid on a global scale. To support the efforts in the creation of novel antifungal drugs, various strategies have been explored to identify new molecular targets in *Malassezia* and possible natural medicines as drug candidates during *in silico* and *in vitro* screening.

The review considers well-known and novel molecular targets in *Malassezia* and highlights the most promising, such as ABC-transporters, lipases, and carbonic anhydrases, for further evaluation. Moreover, the review includes actual and available research data of natural products including plant extracts, essential oils and their molecules on mentioned targets (**Supplementary Table 1**). Based on *in silico* and *in vitro* findings, it is plausible to hypothesize that natural medicines as drug candidates could provide significant antimicrobial effect on scalp microbiota with low incidence of *Malassezia* resistance. They could be used alone or as boosters of currently available first-line synthetic medications to strengthen efficacy because of specific activity on molecular targets and additional beneficial pharmacological effects. The complex approach of using combination of natural drug candidates targeted at several proteins or enzymes could balance between efficiency and good dermatological tolerance in comparison to existing azole drugs. The valuable insights hold great promise for widespread use in cosmetic products and pharmaceutics.

However, this hypothesis requires rigorous validation and persuasive proof-of-concept. Firstly, a major translational challenge lies in the fact that most of the data originates from studies, which do not fully recapitulate the complex scalp microenvironment including high sebum production, pH changes, biofilm formation, specific fungi-bacteria interactions, a thick stratum corneum, frequent washing with surfactant-based shampoos, and the presence of hair follicles that create complex penetration pathways. The scalp is a highly lipophilic environment with a unique composition of saturated and unsaturated fatty acids which serve as sources for *Malassezia* fungi growth. Most of the reviewed compounds (1, 8-cineole, thymol, carvacrol, eugenol, geraniol, xanthomicrol, rosmarinic acid) possess moderate to high logP values of 2–5, which is helpful for penetration into sebum and the lipid-rich stratum corneum as well as into hair follicles via transfollicular route of penetration. However, the high lipophilicity of substances may not only facilitate retention within the superficial layers of the epidermis, but also contributes to sequestration in the sebaceous film, potentially reducing their free concentration at the fungal surface and necessitating higher applied doses to achieve therapeutic efficacy. Moreover, most active natural candidates are washed off within minutes of application on scalp in shampoo composition, severely limiting contact time and compromising drug delivery to the targets of *Malassezia* fungi. Considering this crucial therapeutical point, the concurrent use of topical products like lotions, serums, or creams for scalp is probably essential to maintain prolonged release and antifungal action on *Malassezia* targets for optimal clinical outcomes. Importantly, deep penetration of natural product candidates into hair follicles is being considered as necessary approach, as specific strains of *Malassezia* fungi, such as *Malassezia folliculitis*, could overgrowth in the hair follicles under specific circumstances. Consequently, cosmetic or pharmaceutical formulations should be developed to maximize residence time on scalp rather than to achieve deep transdermal delivery, which also minimizes systemic absorption and reduces the risk of unknown adverse effects because of constant and repeated application. Several of the reviewed terpenes (1, 8-cineole, terpinen-4-ol, eugenol) are known as penetration enhancers that can transiently facilitate penetration to the intercellular lipid domains where *Malassezia* fungi actively growth. Ultimately, a thorough consideration of these scalp-specific physiological parameters, along with rigorous dose-finding studies, is indispensable to bridge the gap between promising *in vitro* findings and reliable clinical outcomes.

Additionally, there is a lack of data of the safety profiles of the 18 natural candidates on the *in vivo* bioavailability, skin penetration, thorough repeated-dose toxicological assessment and skin sensitization. While several components have detailed safety status for topical use, other natural candidates have only fragmentary data or require caution. Among essential oils and their major constituents, the most detailed information exists for *Melaleuca alternifolia* (tea tree) leaf oil, 1, 8-cineole, terpinen-4-ol, and bisabolol, which are generally recognized as safe at typical concentrations up to 5% in the formulations for the prophylaxis and treatment of SD, although oxidized tea tree oil may possess sensitizing potential because of oxide radicals. Conversely, eugenol, thymol, carvacrol, and geraniol are known as substances with skin sensitization potential with restrictions of use at low concentrations lower than 0.1% in topical products according to actual EU regulatory guidelines. Regarding natural plant extracts, *Rosmarinus officinalis* extract and its rosmarinic acid demonstrate promising anti-inflammatory properties to be considered safe, whereas *Cordyceps sinensis* extract shows low irritant potential, but its isolated bioactive compounds such as cordycedipeptide A and cordyceamides have exhibited significant cytotoxicity toward various mammalian cells, underscoring the need for rigorous standardization and purification. The systematic toxicological data for *Acalypha communis* extract, *Solanum atriplicifolium* extract and *Piper crocatum* extract are strictly limited. Importantly, most of the reviewed natural substances are permitted in cosmetic products under current EU regulations and are widely used in personal care formulations with limited dosages. Because of comprehensive phytochemical composition, the reviewed plant extracts and its individual compounds possess a variety of useful therapeutical effects, e.g. antioxidant, anti-inflammatory, antinociceptive, anti-allergic and others. To claim the safety of natural products in comparison with synthetic antifungals, comprehensive comparative scientific evidence remains insufficient for most candidates, especially preclinical evaluation of active concentrations, systematic cytotoxicity testing on relevant skin cell lines (keratinocytes and fibroblasts), research of cumulative antifungal resistance and long-term data on skin irritation and sensitization. Therefore, translation of the natural products into clinical practice necessitates thorough dose-finding studies, standardization of plant extracts, and formulation strategies that minimize irritation and allergic potential as well as comparative toxicological evaluation using actual recognized cell models and OECD guidelines.

Consequently, the direct extrapolation of these findings to clinical efficacy remains speculative at this stage. Convincing proof-of-concept requires extended clinical trials on patients with SD of various severity to establish appropriate dosing regimens, efficacy endpoints, and long-term safety. As also noted for specific plant extracts, the standardization with the development of precise and reproducible analytical methods is crucial and should be performed carefully before moving forward into clinical practice. The use of advanced drug delivery systems (e.g., lipid nanoparticles, microemulsions) would be valuable to improve the bioavailability, stability, and dermatological safety of natural compounds on the scalp. To summarize, the current data about natural drug candidates is not sufficient to support full-scale therapy of SD. Nevertheless, this review provides a solid basis and a rationale for further investigations in this field.
