# Supplementary material for: Novel molecular targets and natural drug candidates for the antifungal therapy of seborrheic dermatitis
Source: Front Cell Infect Microbiol. 2026 Oct 1;16:1813026. doi: 10.3389/fcimb.2026.1813026 (PMC13670024; doi:10.3389/fcimb.2026.1813026)
Supplement: Supplementary file 1 [file Table1.docx]

Supplementary Material

Table 1. Natural product candidates for therapy of SD: molecular targets, evidence levels, key activity data and references

| **Natural product candidate** | **Molecular target** | **Evidence type** | **Key inhibition data and tested fungi species** | **References** |
| --- | --- | --- | --- | --- |
| *Melaleuca alternifolia* leaf oil (tea tree oil) | Lanosterol-14α-demethylase (CYP51) | *in silico*, *in vitro* | Synergistic antifungal effect; growth reduction up to 99.85% *in vitro* (*M. furfur*) | Filatov et al., 2023; Filatov et al., 2024 |
| 1,8-Cineole | Lanosterol-14α-demethylase (CYP51) | *in silico*, *in vitro* | Component of synergistic mixture with tea tree oil and bisabolol (*M. furfur*) | Filatov et al., 2023 |
| (-)-α-Bisabolol | Lanosterol-14α-demethylase (CYP51) | *in silico*, *in vitro* | Component of synergistic mixture; enhances penetration and lanosterol-14α-demethylase inhibition (*M. furfur*) | Filatov et al., 2023 |
| *Piper crocatum* extract (containing erigeside II / dictamnoside A) | Lanosterol-14α-demethylase (CYP51) | *in silico*, *in vitro* | MIC of 1.25–2.5% w/v; binding affinity superior to some azole (*C. albicans)* | Siswina et al., 2022 |
| *Acalypha communis* extract | ABC transporters (MDR1, CDR1) | *in vitro* | Reverses fluconazole resistance (*Candida* spp.) | Gil et al., 2022; Cappiello et al., 2020; Villaescusa‑González et al., 2026 |
| *Solanum atriplicifolium* extract | ABC transporters (MDR1, CDR1) | *in vitro* | Reverses fluconazole resistance (*Candida* spp.) | Gil et al., 2022; Alsherbiny et al., 2015 |
| *Piper longum* (Linn.) extract | Lipase | *in vitro* | Lipase inhibition of 35.32% in a dose‑dependent manier (*M. furfur* enzymatic assay) | Gomare et al., 2018 |
| *Ficus benghalensis* (Linn.) aerial roots extract | Lipase | *in vitro* | Lipase inhibition of 32.18% in a dose‑dependent manier (*M. furfur* enzymatic assay) | Gomare et al., 2018 |
| *Phyllanthus emblica* (Linn.) fruit extract | Lipase | *in vitro* | Lipase inhibition of 26.62% in a dose‑dependent manier (*M. furfur* enzymatic assay) | Gomare et al., 2018 |
| Eugenol | Phospholipase | *in vitro* | Inhibition by 62.3% for 1 hour, 56.6% for 3 hours and 88.7% for 6 hours consequently (*M. pachydermatis*) | Čonková et al., 2024 |
| Terpinen-4-ol | Phospholipase | *in vitro* | Inhibition by 56.6% for 1 hour, 52.8% for 3 hours and 88.7% for 6 hours consequently (*M. pachydermatis*) | Čonková et al., 2024 |
| Thymol | Phospholipase | *in vitro* | Inhibition by 56.6% for 1 hour, 88.7% for 3 hours and 88.7% for 6 hours consequently (*M. pachydermatis*) | Čonková et al., 2024 |
| Carvacrol | Phospholipase | *in vitro* | Inhibition by 26.4% for 1 hour, 73.6% for 3 hours and no growth after 6 hours consequently (*M. pachydermatis*) | Čonková et al., 2024 |
| Geraniol | Phospholipase | *in vitro* | Inhibition by 41.5% for 1 hour, 49.1% for 3 hours and 88.7% for 6 hours consequently (*M. pachydermatis*) | Čonková et al., 2024 |
| *Cordyceps sinensis* mycelia extract (CME-1) | Sphingomyelinase (neutral and acid) | *in vitro* | IC₅₀ ≈ 50 μg/mL for both neutral and acid sphingomyelinases – not tested directly on *Malassezia* | Yuan et al., 2022; Chen et al., 2024; Lee et al., 2020 |
| Xanthomicrol (from *Origanum dictamnus* or *Thymus vulgaris*) | Carbonic anhydrase (β-CA, MgCA) | *in vitro* | *K*i = 0.6 μM selectively for MgCA (*M. globosa* recombinant enzyme) | Paloukopoulou et al., 2022 |
| Rosmarinic acid (from *Thymus vulgaris* or *Rosmarinus officinalis*) | Carbonic anhydrase (β-CA, MgCA) | *in vitro*, *clinical* | *K*i = 2.2 μM selectively for MgCA; clinical trial vs 2% ketoconazole (*M. globosa* recombinant enzyme) | Paloukopoulou et al., 2022; Sadati et al., 2024 |
| *Rosmarinus officinalis* leaf extracts and metabolites (carnosol, carnosic acid) | Aryl hydrocarbon receptor (AhR) in keratinocytes | *in vitro* | >90% inhibition of AhR activation by *Malassezia* ligands FICZ, IND, PZ at 10–100 ppm (human keratinocytes) | Kallimanis et al., 2022 |
